# Anti-Bullying Measures and Initiatives in an Online Setting: Educator Survey

**DOI:** 10.3390/ijerph22040480

**Published:** 2025-03-24

**Authors:** Eric Landers, Juliann Sergi McBrayer, Summer Pannell, Richard Cleveland, Deidre Daniels, Monika Krah

**Affiliations:** 1 Department of Elementary and Special Education, Georgia Southern University, Statesboro, GA 30458, USA; 2 Department of Leadership, Technology, and Human Development, Georgia Southern University, Statesboro, GA 30458, USA; jmcbrayer@georgiasouthern.edu (J.S.M.); rcleveland@georgiasouthern.edu (R.C.); mk16401@georgiasouthern.edu (M.K.); 3 School of Education, The University of Southern Mississippi, Hattiesburg, MS 39406, USA; summer.pannell@usm.edu; 4 Curriculum & Instructional Programs, Georgia Cyber Academy, Atlanta, GA 30349, USA; ddaniels@georgiacyber.org

**Keywords:** bullying, online, virtual schools, cyberbullying

## Abstract

The increased opportunities in virtual schooling offer new opportunities for students but also present new challenges for educators. As virtual enrollment has grown, concerns about student engagement, academic preparedness, and social risks have also grown. Among these concerns is the potential for bullying in online educational settings. While traditional bullying research has been well-documented, studies focusing on bullying within virtual schools remain limited. This study examines teachers’ perceptions of bullying in online schools through a self-reported survey. A total sample of 97 educators from a virtual school was sampled, of which 91% were female. Findings indicate that while physical bullying is rare in the virtual setting, verbal, relational, and cyberbullying are reported by educators. Physical appearance, either body or clothing, was the most reported reason across all types of bullying. Educators reported feeling moderately prepared to identify and respond to these bullying behaviors, but gaps in training and awareness still exist. The study highlights the need for professional development, enhanced digital monitoring, and proactive bullying strategies to fit the virtual learning environment.

## 1. Introduction

The concept of virtual schooling emerged in the mid-1990s when a few non-profit collaboratives and some states began offering students the opportunity to complete their education without attending traditional brick-and-mortar classrooms. These early programs offered fully online programs and supplementary options to help students catch up or complete credits [1]. Since then, online education at the K-12 level has seen significant growth. According to the National Center for Educational Statistics [NCES], between the 2013–2014 and 2019–2020 school year, total virtual school enrollment in the United States rose by over 46% [2]. The number of completely virtual schools increased by 44% during this time [3,4]. While enrollment in the elementary setting declined slightly by 0.83%, enrollment in the middle and high school settings jumped 455% and 105%, respectively. The COVID-19 pandemic during the 2019–2020 school year was a major catalyst for this trend. From the 2019–2020 to 2022–2023 school years, overall virtual enrollment increased by more than 91% in the United States, with elementary school enrollment increasing by 362%, middle school increasing by 90%, and high school increasing by 63% [2]. Overall, from the 2013–2014 to the 2022–2023 school year, total virtual school enrollment increased by over 180% [2].

The expansion of virtual learning options and the COVID-19 global pandemic triggered a shift to online learning that left many educators unprepared [5,6] and increased students’ risk for cyberbullying [7]. As more instruction has turned to the online environment, teachers are unprepared to teach and manage students in this new medium. Effective student engagement and adapting the curriculum for the online environment has been a primary concern [5,6,8,9]. For example, Daniels et al. [5] found that teachers are not prepared to appropriately use questions that would facilitate learning in the online environment. Other researchers have found that teachers and students in the online environment also struggle with device and connectivity issues [10], disparities between students’ homes in terms of student resources for support [10,11], a lack of pedagogical support for teachers [6], and the lack of autonomy and/or work/life balance that comes with working remotely [6]. However, in addition to academic ramifications, the shift to virtual schooling during COVID-19 exposed students to increased potential social risks in online educational settings, including bullying.

### 1.1. Students and Online Learning

Previous research on potential behavioral concerns in the K-12 virtual setting has been limited, with most studies focusing on the academic delivery of content or on behaviors in virtual instruction [5]. However, some researchers have started to examine how the shift to virtual instruction affects social interactions for K-12 students. One major area of concern is the impact on social connections. Many teachers are concerned that students lose personal connections in the virtual environment. Leech et al. [8] found that teachers struggled to maintain connections with students, expressing that the lack of face-to-face interaction made establishing and maintaining relationships difficult. One teacher said, “This is incredibly disconnected and the learning doesn’t feel authentic to them most of the time” [8].

Bakaniene et al. [11] highlighted concerns about the social isolation of students during virtual learning, especially those with disabilities. The lack of in-person interactions with teachers and other students contributed to increased feelings of loneliness. This outcome for students in the virtual setting can be concerning, considering that being a victim of bullying can exacerbate feelings of loneliness and could possibly lead to thoughts of suicide [12,13]. Students who already feel isolated and alone in the virtual setting could be in double jeopardy for bullying and suicidal ideation. Beyond the social implications of feeling isolated, other research has shown that increased virtual presence may also elevate student risk.

Research during the COVID-19 era revealed that K-12 students face potential threats even in the seemingly secure environment of virtual schooling. Hathaway et al. [6] found that teachers had a growing concern for not only their privacy online but that of their students as well. One teacher from the study said, “It is difficult to keep track of students’ schoolwork in the various subjects while also safeguarding privacy considerations in the various digital forums” [6]. Levin [14] underscores the growing threat of cyberattacks on schools, including ransomware attacks, student data breaches, website and social media defacement, and online and school meeting invasions. While Levin reported that just over 1300 publicly disclosed cyber-attack events happened the previous year, the actual number could be 10 to 20 times that many due to issues with reporting. Machusky et al. [10] found that students are directly at risk in the form of email invasions or “Zoom Bombing”, or the act where unauthorized individuals interrupt an online class with hate speech or offensive images. According to Machusky et al. [10], “any learning app with a chat or messaging function can be compromised” (p. 3), posing significant security risks and exposing students to various levels of cyber risks. The rise in cyber risks during virtual schooling has highlighted vulnerabilities that K-12 students might face, leading to increased concerns regarding privacy issues that could contribute to the growing problem of bullying.

### 1.2. Bullying in the Virtual/Online School Setting

While bullying and cyberbullying in the traditional brick-and-mortar setting have been well-documented [15], bullying in the virtual school setting has only begun to receive attention. An early study by Harvey et al. [16] examined bullying in the online setting and found that a very small percentage of students in the online setting (1.4–3.6%) experienced bullying behaviors, such as negative comments, threats, or posting mean or inappropriate things, as compared to the traditional school setting (26.4–59.3%). Since this study in 2014, virtual school enrollment has increased by roughly 180% [2], and the availability of social media has exploded [17]. Furthermore, the percentage of teens who report being online “almost constantly” has doubled, and access to smartphones among teens has increased from 73% to 95%. Much of this online activity occurs without adult supervision [18], leaving students exposed to increased cyber-attacks and bullying. As virtual school enrollment has increased, students face a greater exposure to online harassment.

Recent studies on bullying in the virtual school setting are limited, but they suggest that behaviors have become increasingly complex compared to earlier research. For example, in 2014, Harvey et al. [16] examined behaviors such as rumor-spreading, making negative comments, or feelings of humiliation based on appearance, actions, or clothing, which mirror other researchers’ questions about bullying in traditional settings. However, more recent research suggests that questions used to assess bullying in the virtual school setting should differ from those used in the traditional setting. That is, while students still engage in negative or hurtful comments through chat, the online and disconnected nature of a virtual school allows different opportunities for harassment [18]. For example, these researchers found that some students would upload pictures without permission, negatively and inappropriately alter photos using editing software, or log into other students’ social media accounts to post fake content. Similarly, Mičková et al. [19] identified that students were experiencing and engaging in behaviors specific to the online setting, including instances of account hacking/abuse, password elicitation, and personal data theft as forms of harassment. Given the increasingly complex forms of harassment in virtual schools, there is a pressing need for more specific assessments of varied bullying behaviors in this digital environment.

What is unclear in the rising age of virtual education is to what degree bullying occurs in the online school environment and how prepared educators are to recognize and respond to various types of bullying. What is clear, however, is that as the virtual setting continues to grow, measures need to be taken to identify and intervene before bullying can have an adverse effect on students. In the traditional setting, research has shown that bullying can have a multitude of negative impacts on students, including lower academic achievement [20,21,22], decreased self-esteem [23,24], and increased depression and anxiety [25,26], among an array of other negative outcomes. Research has shown that bullying can be prevented if approached with the goal of establishing a positive school environment and in a proactive manner [27,28,29].

The question is, are adolescents still at risk when there are no physical interactions and learning is limited to the virtual setting? For this study, the researchers support the idea that, like in brick-and-mortar schools, this is indeed happening in the online setting, and schools need to make a concerted effort to keep all kids safe and free from bullying. Some researchers suggest that “the Internet platform may extenuate existing behaviors; traditional bullies can now tap into the ample opportunities afforded via cyberspace to torment victims” [30]. Online disinhibition, or the “lowering of behavioral inhibitions in the online environment” [31], plays a large role in expanding the scope of influence for some bullies, and this phenomenon has been documented in studies of cyberbullying. Furthermore, given the growing number of platforms that encourage online disinhibition, it can come as no surprise that peer victimization beyond the school grounds is increasing. Peer victimization can manifest online in ways “that apparently would not be exhibited in a similar scenario in the ‘real world’” [31]. In other words, because the digital worlds are seen as ‘not real,’ those participating in them do not allocate the same level of self-restraint or concern for the other that would be expected in more real-life arenas. Thus, this high prevalence of bullying paints a threatening picture concerning the safety of youth, especially as school safety incidents continue to rise at a more frequent rate throughout the United States [32]. With more students choosing to attend online schools, a stronger and more in-depth understanding of how bullying plays out in this setting is essential.

## 2. Methodology

### 2.1. Instrument

The “Educator AntiBullying Survey” is a work in collaboration with an institutional center that advocates for youth. It is a 30-question survey that includes five parts and asks educators about physical, verbal, relational, and cyberbullying in their setting. The first four parts ask educators how much they observe each type of bullying, how problematic they perceive each to be, and how prepared they feel to address every kind of bullying. Each of the first four parts presents each type of bullying and questions separately. The fifth part is demographic questions. Content validity was achieved through an extensive literature review and a review of the survey items by a panel of experts in the field. Face validity was achieved by piloting the survey with educators in the field to provide comments on the clarity of the survey items. Minor revisions to the wording were suggested, indicating that the target audience easily understood the items and the questions asked what was intended by the researchers.

### 2.2. Participants and Setting

Great Charter Academy (GCA), pseudonym, is a public K-12 online school in the southeastern region of the United States. GCA serves a statewide (urban, suburban, rural) attendance zone, offering the services and amenities of a traditional brick-and-mortar school without the building. Students access lessons and live classes via an online learning management system and are partnered with certified teachers who instruct and guide student progress and achievement. The survey was sent to 1078 potential participants, of which 108 were administrators, 566 were teachers, and 404 were support personnel.

### 2.3. Data Collection

The Educator AntiBullying Survey was delivered electronically via Qualtrics™, an online survey platform. Before contacting potential participants and administering the survey, permission was granted from the researchers’ Institutional Review Board (IRB) and the school district Superintendent. Contact with potential participants occurred through email as the survey was distributed electronically. Creswell and Creswell (2018) suggested a four-part survey request to include an advance notice alerting potential survey participants, requesting participation in the survey, a follow-up notice, and personalized contact to all non-respondents [33]. The researchers sought a 30% response rate, as a recent study found the average response rate for online empirical studies was 34.2% [34]. The typical completion time for the Educator AntiBullying Survey was 10–15 min. Taking these recommendations into account, along with the need to obtain a high response rate, a four-part invitation to the survey was employed over four weeks. First, a recruitment and advance information email was sent to all potential participants explaining the details of the study and confirming the correct contact information. Second, after one week, an invitation to survey email was sent to all potential participants requesting participation. The invitation to survey email indicated the purpose and significance of the research, anonymity assurance, implied consent, and a link to the survey using Qualtrics™. The invitation to the survey email explained that the survey was anonymous and of a voluntary nature and that all data would be unidentifiable to each participant. Additionally, the invitation to survey email outlined the participant’s rights, including the right to opt out of the survey after having started their responses and the right to skip over questions during the survey. One week following the invitation to the survey email, a third email was sent to remind and follow up with potential survey participants. The researchers sent a fourth email one week later as a final reminder. The survey closed one week after the final reminder email.

### 2.4. Data Analysis

Data analysis was conducted using descriptive statistical methods to summarize participant demographics, bullying behaviors, perceived causes, and teacher preparedness. Frequencies were used to categorize participant demographics, while mean scores were used to quantify the frequency (1 = “0 Times” and 5 equals “six + Times”) and perceived severity of bullying behaviors (1 = “Not Problematic” and 5 = “Very Problematic”) on a five-point Likert scale. Preparedness measures were analyzed using mean scores on a four-point scale, and training history was assessed using a binary response scale (yes/no). The data were coded and analyzed using statistical software to ensure accuracy and consistency in reporting.

## 3. Findings

Table 1 summarizes the sample of 97 participants. The majority of respondents were teachers (75.3%), with smaller representations of support staff (20.6%), administrators (3.1%), counselors (1.0%) and other roles (1.0%). Nearly all participants (98%) worked primarily in an online setting. The sample was predominately female (90.7%) and Caucasian (78.4%). There was almost equal representation across categories regarding years of experience and grade-level setting. Overall, the sample reflects Caucasian, female, teacher-focused participants from an online school setting; see Table 1.

Table 2 analyzes the behaviors described by the bullying category and compares the frequency they are observed with how problematic teachers perceive them to be. Observed is reported in mean scores from a five-point scale where one equals “0 Times” and five equals “six + Times”, whereas problematic is reported as one equals “Not Problematic” and five equals “Very Problematic”. Overall, physical bullying, such as pushing, kicking, and throwing objects, is rarely observed by teachers, with mean frequencies close to 1.0. However, these behaviors are perceived as more problematic, with ratings for behaviors like throwing objects ranging up to 4.0 and 5.0. Verbal bullying, such as teasing (mean 1.46), name-calling (mean 1.55), and inappropriate comments (mean 1.81), occurred more frequently than physical bullying. Relational bullying, like social exclusion and spreading rumors, is observed with means ranging from 1.03 to 1.37. Their problematic ratings indicate that, while observed less, relational bullying is recognized as a significant issue. Like physical bullying, cyberbullying means are low, with some being close to 1.0. However, their problematic ratings are higher, with some behaviors, such as posting harmful content, reaching a maximum score of 5.0. Across all categories, all behaviors tend to be observed less frequently, with their means near the minimal value. However, their perceived problematic nature is more pronounced; see Table 2.

Table 3 describes teachers’ perceptions of why students are bullies across the four types of bullying. Overall, most teachers indicated they were unaware of the reason for physical bullying in their setting. Verbal bullying was the type for which teachers had more clarity about why bullying was occurring. The area teachers noted the most was physical appearance. Taken together, body and clothing appeared to be an area that teachers most often attributed as the reason for bullying behaviors. Notably, across all bullying types, reasons such as race or skin color, religion, sexual orientation, and disability were cited with less frequency, each accounting for less than 8% of responses; see Table 3.

Table 4 describes teacher preparedness to identify and respond to the four types of bullying and their training history. Preparedness is reported in mean scores from a four-point scale where one equals “Very Unprepared” and four equals “Very Prepared”. For identifying bullying, teachers reported the highest mean preparedness for verbal bullying (mean 3.59), followed by relational (3.32), cyberbullying (3.32), and physical bullying (3.19). Similar trends were observed for responding to bullying, where verbal bullying had the highest preparedness mean (3.55). For training, fewer than half of the teachers (41.3%) reported training for physical bullying compared to other types, with most teachers receiving training in cyberbullying (62.5%). Overall, the data suggest that teachers feel prepared to identify and respond to all types of bullying but received less training in identifying physical bullying; see Table 4.

## 4. Discussion

Enrollment in virtual schools has been steadily increasing for the past decade [2]. While virtual schools may offer students increased opportunities to access different teachers and experiences from around the world than their neighborhood brick-and-mortar schools could offer, these settings also bring about new and possibly more complex challenges for teachers. Historically, bullying has been a face-to-face social issue that was primarily perpetrated in schools and neighborhoods [15]. With the proliferation of the internet and social media, bullying transformed from a primarily face-to-face behavior to a cyber activity impacting students beyond the school walls [35]. With more and more students opting for online education, teachers report that bullying has found its way into these new virtual classrooms [18,19,36,37], albeit not in the same form as face-to-face interactions. Some researchers suggest that “the Internet platform may extenuate existing behaviors; traditional bullies can now tap into the ample opportunities afforded via cyberspace to torment victims” [30]. However, despite more students attending virtual schools, research examining bullying in these settings is minimal, and there are still many unknowns regarding what students face. To address this gap in the research, the current study examines teachers’ perceptions of how frequently bullying is observed in the online classroom setting, how problematic they perceive these behaviors to be, and how prepared they are to recognize and address bullying behaviors.

Findings from the current study highlight that while bullying behaviors may not be as prevalent in the virtual classroom setting due to the physical separation, teachers report that these behaviors do occur. This research highlights several significant issues for online education. First, consistent with previous research [18,19,37], verbal bullying, particularly inappropriate comments and name-calling, was among the most observed behaviors and rated the most problematic. The prevalent nature of this type of bullying is similar to traditional brick-and-mortar schools [38,39]. However, unlike traditional schools, verbal bullying in a virtual setting does not always have to be spoken; it can be direct communication in the form of an email or instant message. Cziboly et al. [18] speculated that the impetus of some verbal bullying or escalating verbal interactions was due to misunderstandings in messages sent back and forth between students. That is, the lack of face-to-face context that might include verbal inflections to indicate a joke or a facial expression that shows one person is not serious can often lead to increased misinterpretations of the intended meaning of a message [40], causing increased conflict among students. Another reason could be online disinhibition, or the “lowering of behavioral inhibitions in the online environment” [31]. In other words, a student may have less restraint when responding to a misunderstanding with another peer because they may not see the virtual world as “real” and, therefore, do not realize the harm their words may be causing another. These instances of unresolved miscommunication or verbal bullying could potentially lead to instances of relational bullying.

Second, the low reported frequency of certain bullying types may indicate underreporting, a lack of understanding or knowledge of the students’ online behaviors or interactions, or a misunderstanding of what constitutes bullying in the virtual setting. Physical bullying was rarely observed, which could be because students are not in the same physical space. However, in a setting where one could expect to see higher levels of online interactions and potential cyberbullying [41], teachers in the current study rarely observed this type of bullying. This could be due to a lack of understanding regarding what constitutes this behavior. Early definitions of cyberbullying differentiated it from traditional bullying by highlighting the anonymity of the behavior and focusing on repeated acts through sending mean messages or spreading rumors in an online format [42]. However, cyberbullying has evolved past the simple mean text that characterized it two decades ago and morphed into visual or sexual harassment (sharing explicit content), character impersonation (fake identities or catfishing), and digital exclusion (leaving someone off text threads or not “friending” them) [43]. In the current study, cyberbullying, although reported as less frequent, is perceived as highly problematic when it does occur. This could suggest that there are unique challenges associated with bullying in virtual schools and that teachers may not be aware of the current cyberbullying tactics that students are using in the virtual world.

Finally, the current study examined teachers’ varying perceptions of why bullying occurs. The majority of the teachers reported that they were uncertain about the reason for the bullying, particularly regarding physical and cyberbullying. Among the identified reasons, physical appearance (body and clothing) was the most frequently cited cause for bullying across all types. Other reasons, including race, sexual orientation, religion, and disability, were reported less often, which may suggest that these are underreported or not as visible in the virtual setting. These findings are similar to previous studies that found that students are primarily bullied because of perceived differences [44]. However, the high uncertainty about the causes of bullying does indicate a need for training to equip teachers to recognize and respond to these behaviors.

### 4.1. Implications for Practice

The current study highlights several implications for practice. First, teachers have historically reported the need for training in identifying and addressing bullying [45,46], and research shows that bullying training can increase awareness of bullying and improve the school environment [47]. The current findings highlight this need for teachers in the virtual setting and suggest teachers would benefit from in-depth training that addresses the complexities of recognizing and responding to bullying in virtual settings. This training should focus on training teaching to identify the forms of bullying specific to the virtual setting, which are often less visible but have psychological and emotional impacts on students. Second, given that much of the bullying in online schools is more difficult to detect because it is not as overt as in the traditional setting, schools can implement comprehensive monitoring and reporting systems that use digital tools, such as chat logs, to detect bullying early so intervention can be provided before too much harm is done. Third, research has shown that bullying can have a long-term cumulative impact on students [48]; therefore, schools should take a proactive approach to prevent bullying. The virtual setting is essential to promote digital citizenship, foster a culture of respect among students, and encourage positive engagement. Finally, schools can also engage parents in the process by educating them on how to monitor their child’s online activity in the virtual environment and suggest ways to address and report instances of suspected bullying.

### 4.2. Recommendations for Future Research

Future research should focus on several key areas to address gaps in the research involving bullying and student interactions in the virtual school environment. Since very little research has been conducted on bullying in this setting, future research should focus on longitudinal studies to examine long-term bullying trends in virtual school environments and how teachers can implement sustainable, effective interventions. It is also critical for future research to explore the experiences of marginalized populations, such as students with disabilities, racial minorities, and LGBTQ+ students, who may face unique forms of bullying in online spaces. With rates of bullying increasing for many of these populations [49], understanding how these groups are impacted can inform the development of more inclusive practices and provide additional support. Finally, future studies should examine how advanced technologies, such as artificial intelligence, can detect and mitigate bullying behaviors in real time, providing immediate support to both targets of bullying and educators.

## 5. Conclusions

As virtual schools continue to grow, it is important to recognize that bullying is not limited to traditional classrooms but is also present in online education. This study highlights the need for greater awareness and proactive measures to address bullying in virtual settings. While teachers acknowledge the presence of online bullying, changing digital behaviors presents challenges in effectively identifying and responding to these issues. Schools should focus on educator training, implementing monitoring strategies, and fostering a positive digital school climate.

## Figures and Tables

**Table 1:** Demographic information.

School Setting	*n*	%
Online	95	97.9
Hybrid	2	2.1
Position		
Teacher	73	75.3
Administration	3	3.1
Counselor	1	1.0
Support Staff	19	19.6
Other	1	1.0
Years of Experience		
0–5	17	17.5
6–10	22	22.7
11–15	21	21.6
16–20	20	20.6
20+	16	16.5
Gender		
Male	8	9.0
Female	88	91.0
Prefer Not to Say	1	1.0
Race		
American Indian or Alaskan Native	1	1.0
Black or African American	20	20.6
White	76	78.4

**Table 2:** Bullying type by frequency versus problematic nature.

	Observed		Problematic	
	Mean	Range	Mean	Range
Physically Bullying				
Pushing and Shoving	1.02	1.0–2.0	1.03	1.0–4.0
Kicking, Hitting, or Punching	1.03	1.0–2.0	1.05	1.0–4.0
Throwing Objects at Other Students	1.02	1.0–2.0	1.05	1.0–5.0
Inappropriate Touching	1.00	1.0–1.0	1.02	1.0–2.0
Inappropriate Sexual Touching	1.00	1.0–1.0	1.02	1.0–2.0
Damaging Someone’s Property	1.01	1.0–2.0	1.04	1.0–2.0
Taking Someone’s Property Without Permission	1.00	1.0–1.0	1.03	1.0–4.0
Verbal Bullying				
Teasing or Taunting	1.50	1.0–4.0	1.35	1.0–4.0
Threat to Harm	1.09	1.0–3.0	1.18	1.0–3.0
Name-Calling	1.55	1.0–5.0	1.56	1.0–5.0
Inappropriate Comments	1.81	1.0–5.0	1.76	1.0–5.0
Inappropriate Sexual Comments	1.18	1.0–3.0	1.32	1.0–5.0
Inappropriate Racial Remarks	1.15	1.0–3.0	1.29	1.0–5.0
Relational Bullying				
Social Exclusion	1.29	1.0–4.0	1.28	1.0–3.0
Unfriending	1.24	1.0–4.0	1.26	1.0–4.0
Spreading Rumors or Gossip	1.13	1.0–3.0	1.20	1.0–4.0
Inappropriate Jokes About Others	1.31	1.0–3.0	1.37	1.0–5.0
Inappropriate Sexual Jokes About Others	1.03	1.0–2.0	1.11	1.0–5.0
Cyberbullying				
Making False Claims or Insults Online	1.28	1.0–3.0	1.30	1.0–5.0
Emailing Harmful Things to Others	1.09	1.0–3.0	1.11	1.0–4.0
Posting Harmful Pictures/Video Online Without Permission	1.08	1.0–3.0	1.10	1.0–4.0
Excluding Someone From Online/Text Activity	1.16	1.0–3.0	1.18	1.0–4.0
Deleting Personal Electronic Information	1.06	1.0–3.0	1.09	1.0–4.0
Hacking/Posting to Someone’s Account	1.07	1.0–2.0	1.08	1.0–4.0
Creating Fake Profiles to Pretend to be Someone Else	1.06	1.0–2.0	1.11	1.0–4.0
Creating Harmful Webpage/Fake Profiles to Harass	1.02	1.0–2.0	1.07	1.0–4.0
Cyberstalking/ Closely Monitoring Another Online	1.04	1.0–2.0	1.11	1.0–4.0
Publishing Someone’s Personal Information	1.01	1.0–2.0	1.07	1.0–4.0
Creating Fake Images Using AI	1.06	1.0–2.0	1.11	1.0–4.0

**Table 3:** Teacher-perceived reasons students are bullied by type.

	Physical Bullying		Verbal Bullying		Relational Bullying		Cyber Bullying	
	*N*	%	*N*	%	*N*	%	*N*	%
I don’t know	75	55.15	41	22.65	59	40.97	59	46.83
Race or skin color	8	5.88	13	7.18	9	6.25	8	6.35
Physical (body)	11	8.09	30	16.57	16	11.11	15	11.90
Physical (clothing)	10	7.35	15	8.29	11	7.64	10	7.94
Disability	4	2.94	10	5.52	10	6.94	6	4.76
Religion	6	4.41	13	7.18	6	4.17	6	4.76
Sexual Orientation	6	4.41	13	7.18	8	5.56	8	6.35
Socioeconomic status (poor)	5	3.68	10	5.52	7	4.86	5	3.97
Socioeconomic status (rich)	2	1.47	4	2.21	2	1.39	3	2.38
Other	9	6.62	32	17.68	16	11.11	6	4.76

Note. Total valid responses for physical bullying were *N* = 136; verbal bullying *N* = 181; relational bullying *N* = 144; and cyberbullying *N* = 126.

**Table 4:** Preparedness for identification and management of instances of bullying.

	Physical		Verbal		Relational		Cyber	
	Mean		Mean		Mean		Mean	
I feel prepared to identify physical bullying in the school setting.	3.18		3.59		3.32		3.32	
I feel prepared to respond to physical bullying in the school setting.	3.07		3.55		3.26		3.29	
Have you received training to respond to bullying?	Yes	No	Yes	No	Yes	No	Yes	No
	41.3%	59.7%	60.7%	39.3%	57.1%	42.9%	62.5%	37.5%

## Data Availability

The data supporting the findings of this study are not available as participants did not provide explicit consent for data sharing. To protect participant privacy and comply with ethical guidelines, the dataset cannot be publicly released.
